# The Generation of Animal Heat in the Lungs

**Published:** 1891-05

**Authors:** A. D. Barr

**Affiliations:** Calamine, Ark.


### THE GENERATION OF ANIMAL HEAT IN THE LUNGS.

BY A. D. BARR, M. D., CALAMINE, ARK.

Lavoisier maintained that the lungs might be considered as the principal centre of the generation of animal heat. This idea was soon proven to be erroneous, since which time the lungs have been credited with the production of a very inconsiderable amount of the total heat produced by the human body. Since such very important chemical changes take place in the lungs, it becomes very interesting to examine into these changes and see if any chemical combinations occur that will account for the production of any important amount of heat. In the study of digestion, we find that the fats are absorbed by the lymphatics, and are poured into the blood current at the junction of the left internal jugular and subclavian veins. From here they pass, as fats, to the heart and thence to the lungs. In the lungs the fats seem to be acted upon by the oxygen, and so changed that they are no longer recognized in their original form. In their transformation they yield heat, which is capable of being converted into other forms of energy. Sugar may also be traced to the lungs, where, ordinarily, it is so decomposed that it can be no longer recognized, and in its decomposition it also yields heat, which is the result of all chemical combinations. The union of the oxygen of the inspired air and the hæmoglobin of the blood is of course followed by the production of heat.

Having seen that important chemical combinations take place in the lungs, it becomes important to see if we can arrive at an approximate idea of the amount of heat there generated. This is best done by considering the mode by which heat is lost in the percentage form. Helmholtz considers that 2.6 per cent. of the total heat is expended in heating the food; 2.6 per cent. of the total heat of the body is expended in heating the air inspired, supposing its temperature to be raised from 20° c. to 30° c.; 14.7 per cent. of the total heat of the body is expended in evaporating the water eliminated by the lungs; 80.1 per cent. of the total heat is expended by radiation, conduction and by evaporation of water from the skin. According to the above $17\frac{3}{10}$ per cent. of the total heat produced by the body is given off by the lungs. The next question is, if no heat is generated in the lungs worth taking into consideration, what is the amount of the total heat that is conveyed to the lungs by the blood? This can best be answered by ascertaining the proportion in which the blood is distributed to the different organs, which, according to Ranke, is, that one-fourth of the total amount of the blood is contained in the muscles, one-fourth in the liver, one-fourth in the heart and great vessels, and one-fourth in the remaining organs. Now the remaining fourth of the blood, according to Ranke, is to be divided between the brain, the spleen, the kindneys, the stomach and intestines and the lungs.

Say, give the lungs one-sixteenth the total amount of the blood and they will contain 16 per cent. of the total heat of the body Then 16 per cent. of the total heat of the body is contained in the lungs, and $17\frac{3}{10}$ per cent. of the total amount of the body-heat is given off by the lungs; thus it is evident that more heat is given off by the lungs than these organs could possibly contain unless a very considerable amount of heat was generated in them. And, again, if no heat was generated in the lungs, the blood returned from these organs, instead of being reduced about one-third of a degree F., would be reduced to zero. For these reasons I am forced to conclude that a very considerable amount of the entire heat of the animal body is generated in the lungs.

